# Tomato glycosyltransferase Twi1 plays a role in flavonoid glycosylation and defence against virus

**DOI:** 10.1186/s12870-019-2063-9

**Published:** 2019-10-26

**Authors:** Laura Campos, María Pilar López-Gresa, Diana Fuertes, José María Bellés, Ismael Rodrigo, Purificación Lisón

**Affiliations:** Instituto de Biología Molecular y Celular de Plantas, Universitat Politècnica de València-Consejo Superior de Investigaciones Científicas, Valencia, Spain

**Keywords:** Glycosyltransferase, *Tomato wound-induced* gene, Coumarins, Flavonoids, Pathogen, Transgenic plants

## Abstract

**Background:**

Secondary metabolites play an important role in the plant defensive response. They are produced as a defence mechanism against biotic stress by providing plants with antimicrobial and antioxidant weapons. In higher plants, the majority of secondary metabolites accumulate as glycoconjugates. Glycosylation is one of the commonest modifications of secondary metabolites, and is carried out by enzymes called glycosyltransferases.

**Results:**

Here we provide evidence that the previously described tomato wound and pathogen-induced glycosyltransferase Twi1 displays in vitro activity toward the coumarins scopoletin, umbelliferone and esculetin, and the flavonoids quercetin and kaempferol, by uncovering a new role of this gene in plant glycosylation. To test its activity in vivo, *Twi1*-silenced transgenic tomato plants were generated and infected with Tomato spotted wilt virus. The *Twi1*-silenced plants showed a differential accumulation of Twi1 substrates and enhanced susceptibility to the virus.

**Conclusions:**

Biochemical in vitro assays and transgenic plants generation proved to be useful strategies to assign a role of tomato Twi1 in the plant defence response. Twi1 glycosyltransferase showed to regulate quercetin and kaempferol levels in tomato plants, affecting plant resistance to viral infection.

## Background

Plants are characterised by their ability to synthesise a wide variety of secondary metabolites that exert multiple and important functions for plants to interact with their environment [1, 2]. The high structural diversity and complexity of these compounds is generated by a number of modifying enzymes, such as methyltransferases, acyltransferases and glycosyltransferases [3]. Phenolic compounds, which derive from phenylalanine, are among the most widespread groups of plant secondary metabolites, and display a wide range of biological properties [4]. The synthesis and accumulation of many plant phenolics are induced by biotic and abiotic stresses [5]. Salicylic acid (SA) is a well-known phenolic compound that is induced by pathogen attack and has been shown to play a crucial role as a phytohormone by regulating the plant response to biotic stress [6]. Biotrophic pathogens, including numerous bacteria, fungi and viruses, are combated mostly by the SA-signalling defence pathway.

Many pathogen-induced phenolic compounds, including various coumarins and flavonoids, are considered phytoalexins given their accumulation in plant tissues upon infection and their antimicrobial activity in vitro [4, 7]. Besides their antimicrobial properties, several phenolics have been shown to display relevant antioxidant activity in vitro [8]. For instance, scopoletin is a well-characterised hydroxycoumarin that is produced in many plant species, and has been proposed as an important phytoalexin against microbial pathogens [9]. Among plant secondary metabolites, flavonoids comprise one of the most abundant and important groups and exhibit a diversity of biological functions [10, 11]. The antioxidant and antifungal activities of various flavonoids, including quercetin and kaempferol, have been reported in several studies [12–15].

Glycosylation is one of the commonest modifications found in plant secondary metabolites [16], which alters the sugar acceptor by reducing its toxicity, increasing its solubility and accumulation, and regulating its subcellular localisation and bioactivity, such as antioxidant capacity [17]. Glycosylation is also involved in the detoxification of xenobiotics and the regulation of the active levels of various hormones [18–20]. Conjugated secondary metabolites generally exhibit diminished chemical activity compared to the aglycone by acting merely as storage and/or transport forms [21].

The majority of glycosylation reactions of secondary metabolites are catalysed by uridine diphosphate (UDP) sugar-dependent glycosyltransferases (UGTs) [22]. The *Arabidopsis* genome contains more than 120 *UGT* genes [23]. In the continuously updated Carbohydrate Active enZymes (CAZy) database (http://www.cazy.org/GlycosylTransferases.html), glycosyltransferases from diverse organisms are currently classified into 105 families. The UGTs belonging to multigene Family 1 predominantly recognise low-molecular-weight compounds, such as phenolics, as substrates [22, 24].

A role in plant disease resistance has been reported for numerous UGTs. In tobacco, *Togt1* and *Togt2*, were induced during a hypersensitive response (HR) and exhibited high efficiency upon hydroxycoumarins conjugation [25, 26]. In addition, transgenic *TOGT*-silenced tobacco plants showed increasing susceptibility to Tobacco mosaic virus (TMV) [27]. Another UGTs, such as *CaUGT1* in *Capsicum annuum* and both *UGT73B3* and *UGT73B5* in *Arabidopsis*, were induced upon TMV and *Pseudomonas syringae* inoculation, respectively [28]. Furthermore, *CaUGT1*-silenced plants showed a delayed HR [29], and studies conducted with *ugt73b3* and *ugt73b5* mutants revealed that both UGTs were essential components to maintain the redox status in resistance of *Arabidopsis* to bacterial infection [30]. Recently, UGT76D1 has been described as a unique glycosyltransferase which plays a key role in SA homeostasis related to immune responses in *Arabidopsis* [31].

Among all the identified UGTs, only a few of them have been biologically characterised. In many cases, in vitro studies have identified secondary metabolites, such as phenylpropanoids and flavonoids, as UGT substrates, while their function in vivo remains elusive [11, 27, 30, 32–37]. To date, available information about the actual *in planta* UGTs substrates is limited [38] and the precise contribution of glycosyltransferases in the plant response to biotic stresses remains unclear [23, 39].

In tomato, some UGTs have been identified as related to biotic and abiotic stresses. GAGT is a gentisic acid (GA) glycosyltransferase induced in tomato upon Citrus exocortis viroid (CEVd) and Tomato mosaic virus (ToMV) infection [35]. Recently, it has been described that SlUGT75C1 catalyzes the glucosylation of abscisic acid in tomato, and its suppression by RNAi produced plants more resistant to drought stress [40].

The tomato wound-induced gene *Twi1* encodes a putative UGT. *Twi1* appears to be induced by exogenously applied SA and its derivatives, and is rapidly expressed during the resistance response to fungal elicitor treatment [41]. However, the biochemical activity and the defensive role of this UGT have not yet been explored.

This study aims to gain insight into the role of Twi1 in the tomato defence response against pathogens. For this purpose, the Twi1 recombinant protein was purified and its activity was assayed in vitro toward a wide range of putative substrates. We have generated *Twi1*-silenced transgenic tomato plants and infected them with Tomato spotted wilt virus (TSWV) to study the resistance phenotype and to analyse metabolite profiling upon infection. Our results indicated that Twi1 could play an important role in the metabolism of defence-related compounds in tomato.

## Results

### *Twi1* induction in response to plant pathogens

The wound-induced gene of tomato *Twi1* has been described to be rapidly induced by SA application and a fungal elicitor [41]. Quantitative RT-PCR (qRT-PCR) confirmed the induction of *Twi1* mRNA by stem-feeding SA treatment, and a rapid decline some hours later. A slight induction of *Twi1* was observed in the control water-treated plants due to the wounding produced by the stem-feeding technique (Additional file 1: Figure S1).

**Fig. 1:**
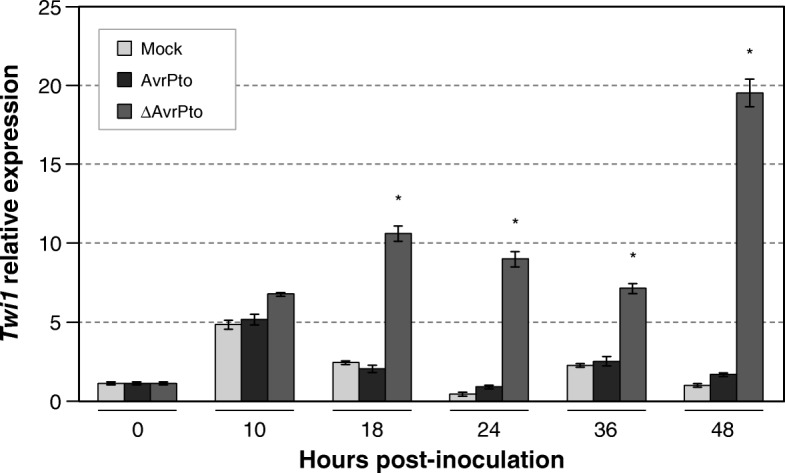
*Twi1* gene expression in tomato after inoculation with *Pseudomonas syringae* pv. *tomato*. The quantitative reverse transcription-polymerase chain reaction (qRT-PCR) analysis of *Twi1* gene expression in the inoculated and Mock-inoculated leaves from tomato plants cv. ‘Rio Grande’ upon inoculation with the *Pseudomonas syringae* pv. *tomato* DC3000 avirulent (AvrPto) or virulent (∆AvrPto) strains. The *Elongation Factor 1 alpha* (*eEF1α*) gene was used as an endogenous reference. The results correspond to the means±SD of three independent plants from a representative experiment. A *t-*test was performed with the data from three independent experiments. Asterisks (*) indicate statistical significance with a *p* value < 0.05 in relation to the Mock-inoculated plants

To study the expression of *Twi1* in response to bacterial infection, tomato plants cv. ‘Rio Grande’ carrying the *Pto* resistance gene were inoculated with a virulent and an avirulent strain of *P. syringae* pv. *tomato* (*Pst*) DC3000 (see Methods). Leaf samples were collected at the indicated time points and analysed by qRT-PCR to quantify the *Twi1* levels during infection (Fig. 1). *Twi1* was strongly induced in the tomato plants inoculated with the virulent bacteria (*Pst* DC3000 ∆*AvrPto*) between 18 h and 48 h post-inoculation*,* while the induction level in the *Pst* DC3000 *AvrPto* inoculated plants was comparable to that observed in the *mock*-inoculated plants. The slight induction observed in both the *mock* and *Pst* DC3000 *AvrPto* inoculated plants could probably be due to the minor leaf damage that occurred during immersion.

**Fig. 2:**
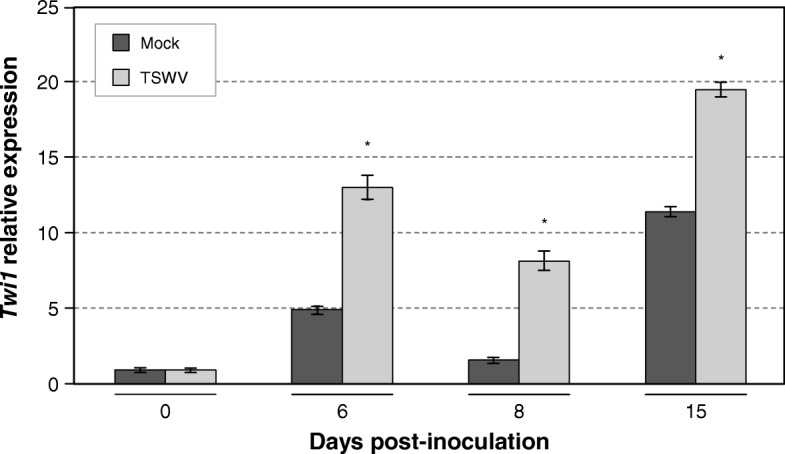
*Twi1* gene expression in tomato after inoculation with Tomato spotted wilt virus (TSWV). A quantitative reverse transcription-polymerase chain reaction (qRT-PCR) analysis of *Twi1* gene expression in the inoculated and Mock-inoculated leaves from tomato plants cv. Moneymaker upon inoculation with TSWV. The *Elongation Factor 1 alpha* (*eEF1α*) gene was used as an endogenous reference. The results correspond to the means±SD of three independent plants from a representative experiment. A *t-*test was performed with the data from three independent experiments. Asterisks (*) indicate statistical significance with a *p* value < 0.05 in relation to the Mock-inoculated plants

The *Twi1* expression in the Moneymaker tomato plants (our control or wild type plants) infected with TSWV was also analysed by qRT-PCR (Fig. 2). Once again, a basal *Twi1* induction in the *mock*-inoculated plants was detected. Since this induction was not only produced at early times but also at 15 days after the virus inoculation, additional factors apart from the mechanical wounding could be involved. Nonetheless, the expression levels were significantly higher in the tomato plants inoculated with TSWV during infection at all timepoints.

### *Twi1* cloning and in vitro enzymatic activity assay

To obtain the full-length cDNA of *Twi1,* PCR amplification was performed from the RNA of the Moneymaker tomato leaves infected with *Pst* DC3000 ∆*AvrPto*, where *Twi1* was highly expressed. The PCR product was cloned in pGWB8, a binary vector for gene overexpression in plants, which added a His-tag to the C-terminus and was then transformed into *A. tumefaciens*. The recombinant protein was transiently expressed in the agroinfiltrated *N. benthamiana* leaves and purified (Additional file 2: Figure S2). The fractions bearing the His-tagged protein were pooled to perform the Twi1 in vitro activity test.

**Fig. 3:**
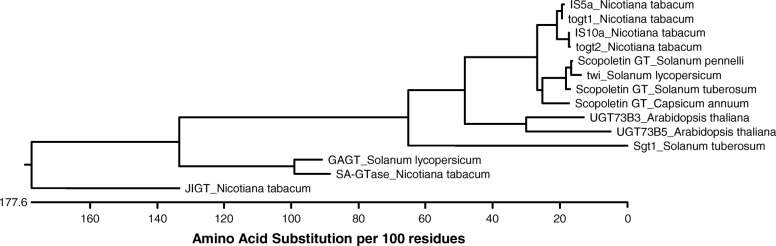
Twi1 phylogenetic tree with different glycosyltransferases involved in plant defence. The glycosyltransferases included in the analysis were IS5a (AAB36653), togt1 (AAK28303), IS10a (AAB36652), togt2 (AAK28304), SA-GTase (AAF61647) and JIGT (BAA19155) from *Nicotiana tabacum*. Scopoletin GT from *Solanum pennellii* (XP_015062099), Twi1 (CAA59450) and GAGT (CAI62049) from *Solanum lycopersicum*, Scopoletin GT from *Solanum tuberosum* (XP_006346388), Scopoletin GT from *Capsicum annuum* (XP_016539537), UGT73B3 (NP_567953) and UGT73B5 (NP_179150) from *Arabidopsis thaliana*, and Sgt1 from *Solanum tuberosum* (AAB48444)

Both the BLAST analysis and a phylogenetic tree based on the full-length sequence of the 13 UGTs involved in plant defence (Fig. 3) suggested that *Twi1* was likely to encode a scopoletin glucosyltransferase, given its close vicinity to the predicted scopoletin UGTs identified in *S. pennellii*, *S. tuberosum* and *C. annuum*.

**Fig. 4:**
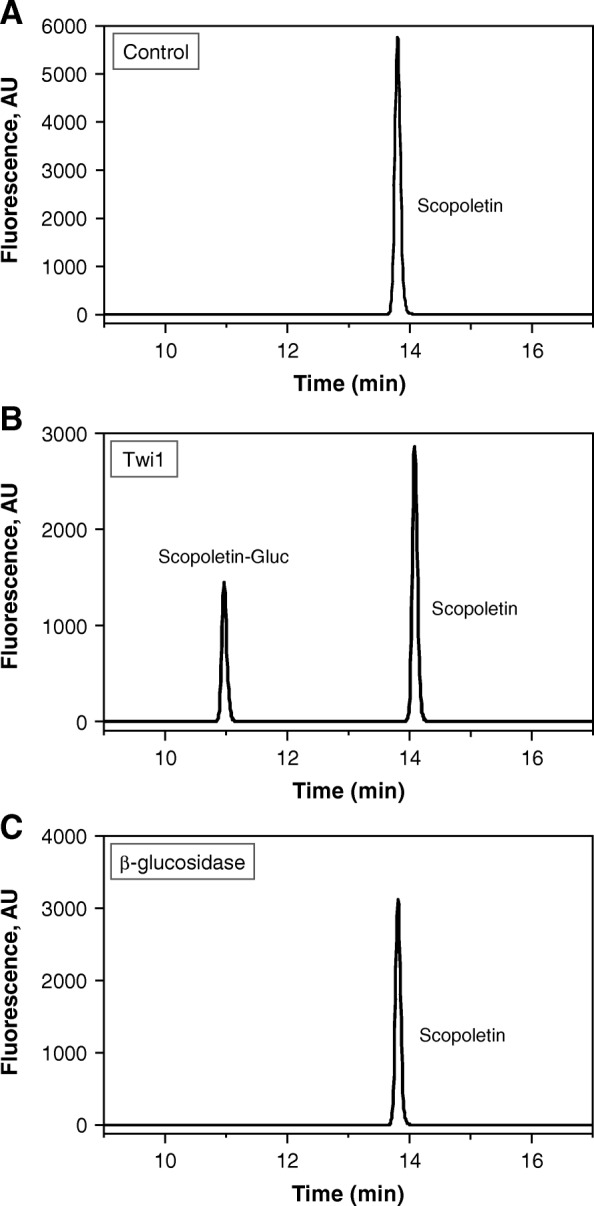
Twi1 in vitro activity assay with scopoletin as the sugar acceptor. **a** The chromatographic peak obtained when scopoletin was incubated with UDP-glucose in the presence of the Control sample (protein extract from the *N. benthamiana* leaves agroinoculated with the pGWB8 empty vector). **b** The chromatogram obtained upon incubation with the Twi1 purified recombinant protein expressed in the *N. benthamiana* agroinoculated leaves. A differential peak was detected, corresponding to scopoletin conjugated with glucose. **c** The product of the reaction shown in (**b**) was incubated with beta-glucosidase. A free scopoletin peak was the only one detected after treatment. Samples were analysed by HPLC-fluorescence

The glycosyltransferase activity of the purified recombinant Twi1 enzyme was assayed against a variety of phenolic sugar acceptors using UDP-glucose as the sugar donor. The compounds displaying positive (+) or negative (−) behaviour as substrates are shown in Table 1. The reaction products were analysed using either HPLC coupled with a fluorescence detector or UPLC-Q-ToF-MS, depending on the nature of both the substrates and products, and in comparison to reference standards. The recombinant protein was able to glycosylate 5 of the 14 substrates tested in vitro: 2,4-DHBA, 2,4,6-THBA, scopoletin, esculetin and umbelliferone. This Twi1 glucosyltransferase activity is exemplified with scopoletin as the sugar acceptor in Fig. 4. The first chromatogram shows a peak, which corresponds to free scopoletin (Fig. 4a), obtained when scopoletin was incubated with UDP-glucose in the presence of the extracts from the control plants. In the presence of the Twi1 purified protein, a differential peak was detected, which corresponded to scopolin, i.e. scopoletin conjugated with glucose (Fig. 4b). To establish the type of linkage between scopoletin and glucose moiety, the reaction product was incubated with beta-glucosidase. An HPLC-fluorescence analysis of the sample revealed that scopoletin was fully recovered in its free form after treatment (Fig. 4c), which indicates that the O-glycoside bond catalysed by Twi1 between glucose and scopoletin was in a beta-position. No bond cleavage was detected when alpha-glucosidase was used (not shown). Regarding sugar donor specificity, UDP-xylose was also investigated, but no glycosylation was detected in any studied substrate. The Twi1 glucosyltransferase activity towards umbelliferone, 2,4-DHBA and esculetin as sugar acceptors is shown in Additional file 3: Figure S3. The activity of Twi1 with 2,4,6-THBA is shown in Additional file 4: Figure S4.

**Table 1:** The Twi1 in vitro glycosyltransferase activity assay. The Twi1 purified recombinant protein was tested for glycosyltransferase activity in vitro using UDP-glucose and the following phenolics as sugar acceptors: 2,4-Dihydroxybenzoic acid (2,4-DHBA), 2,4,6-Trihydroxybenzoic acid (2,4,6-THBA), Benzoic acid, Salicylic acid, 4-Hydroxybenzoic acid, Scopoletin, Esculetin, Umbelliferone, Cinnamic acid, *p*-coumaric acid, Caffeic acid, Ferulic acid, *o*-coumaric acid and Chlorogenic acid. Samples were analysed by HPLC-fluorescence or UPLC-Q-ToF-MS to detect sugar acceptor and/or glycoconjugate formation

Compound	Activity^(a)^	Method for detection
2,4-DHBA	+	HPLC-fluorescence
2,4,6-THBA	+	UPLC-Q-ToF-MS
4-Hydroxybenzoic acid	–	UPLC-Q-ToF-MS
Benzoic acid	–	UPLC-Q-ToF-MS
Salicylic acid	–	HPLC-fluorescence
Scopoletin	+	HPLC-fluorescence
Esculetin	+	HPLC-fluorescence
Umbelliferone	+	HPLC-fluorescence
Caffeic acid	–	UPLC-Q-ToF-MS
Cinnamic acid	–	UPLC-Q-ToF-MS
Chlorogenic acid	–	UPLC-Q-ToF-MS
*o-*coumaric acid	–	UPLC-Q-ToF-MS
*p-*coumaric acid	–	UPLC-Q-ToF-MS
Ferulic acid	–	UPLC-Q-ToF-MS

(^a^) Plus: glycoconjugate detected

Minus: glycoconjugate not detected

To our knowledge, neither the accumulation nor the biological activity of 2,4-DHBA and 2,4,6-THBA has been described in plants. For that reason, we decided to focus our study of the coumarins scopoletin, esculetin and umbelliferone as Twi1 substrates *in planta*, with a putative function in tomato defence against pathogens.

### Generation and characterisation of the *Twi1-*silenced tomato plants

To gain further insights into the role of Twi1 in vivo, the *Twi1*-silenced transgenic Moneymaker tomato plants were generated by following an RNAi strategy.

Regenerated lines were analysed for T-DNA integration by PCR, and nine independent transgenic lines were confirmed. Two homozygous lines, 1.1 and 28.3 both carrying one copy of the transgene, were selected for further studies. Phenotypic parameters were similar for both the RNAi Twi1 transgenic and wild-type (wt) plants.

**Fig. 5:**
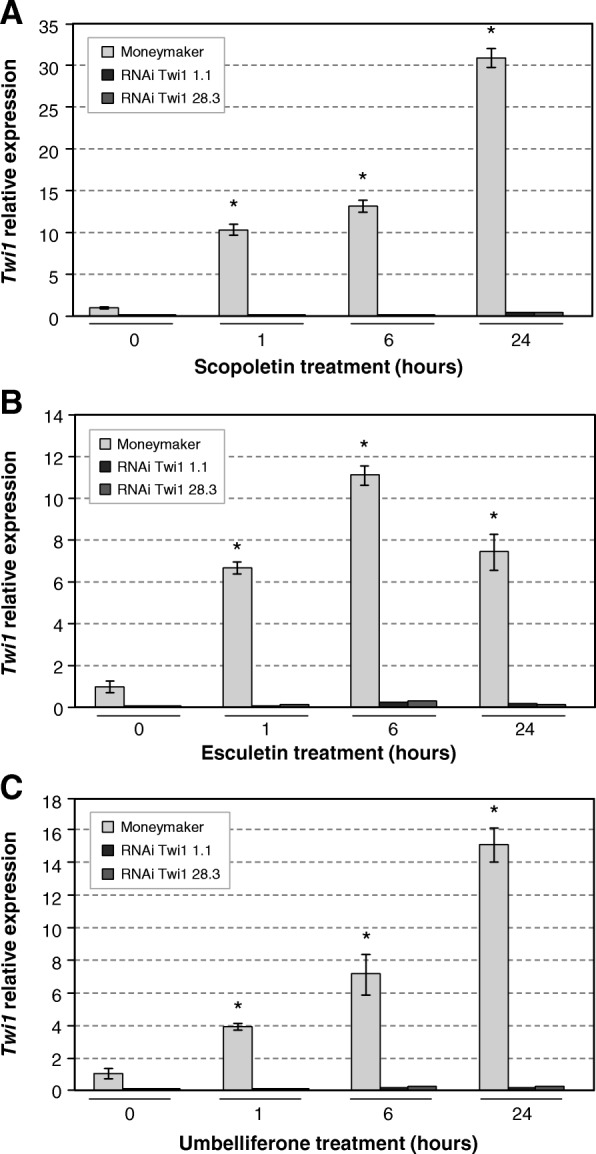
*Twi1* gene expression in the Moneymaker and RNAi Twi1 transgenic tomato upon scopoletin, esculetin or umbelliferone treatment. A quantitative reverse transcription-polymerase chain reaction (qRT-PCR) analysis of *Twi1* gene expression in the leaves of wt (Moneymaker), and the RNAi Twi1 transgenic tomato lines 1.1 and 28.3 upon the scopoletin (**a**), esculetin (**b**) or umbelliferone (**c**) stem-feeding treatments. The *Elongation Factor 1 alpha* (*eEF1α*) gene was used as an endogenous reference. The results correspond to the means±SD of three independent plants from a representative experiment. A *t-*test was performed with the data from three independent experiments. Asterisks (*) indicate statistical significance with a *p* value < 0.05 in relation to the wt plants

The induction of *Twi1* by stem-feeding treatments with in vitro substrates was analysed in both the control and RNAi Twi1 transgenic plants, in order to confirm the biological role of the enzyme. Treatments with scopoletin, esculetin and umbelliferone resulted in a rapid outstanding induction of *Twi1* expression in the wt tomato plants, as shown by the qRT-PCR analysis (Fig. 5).

After a 1-h treatment with scopoletin, the *Twi1* transcript levels increased 10-folds, and continued to rise during the 24-h treatment. Similarly, *Twi1* was also induced by the esculetin and umbelliferone treatments. As expected, a sharp drop in the *Twi1* transcripts was observed in the treated RNAi transgenic lines 1.1 and 28.3 compared to the corresponding wt plants, which confirms the efficacy of the gene silencing strategy. The basal level of *Twi1* detected in the wt plants could be due to the wounding produced in the stem-feeding technique (Additional file 1: Figure S1).

### Analysis of the *Twi1*-silenced transgenic tomato plants upon infection with TSWV

**Fig. 6:**
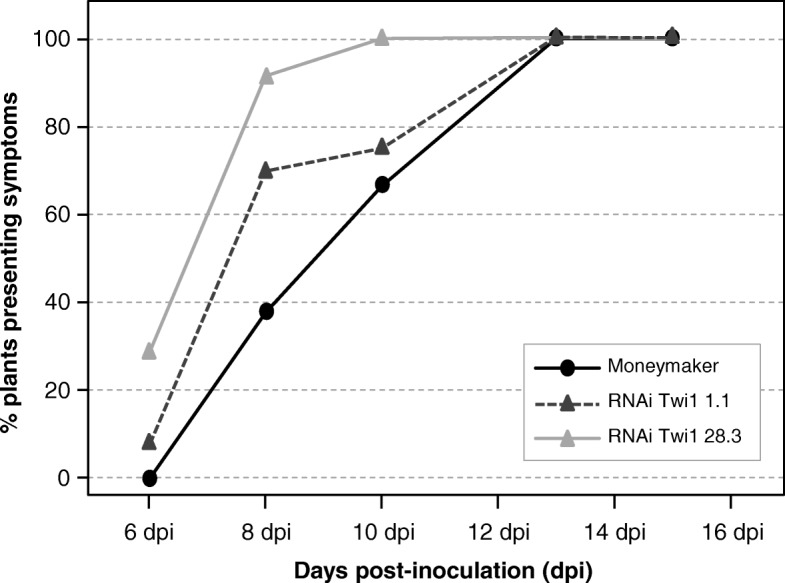
Disease development in the Moneymaker and RNAi Twi1 transgenic tomato inoculated with Tomato spotted wilt virus (TSWV). The Infectivity Index was measured in the RNAi Twi1 transgenic tomato and wt (Moneymaker) plants inoculated with TSWV. The percentage of wt plants (black circles), plants from line 1.1 (dark grey triangles) and plants from line 28.3 (light grey triangles) showing symptoms at 6, 8, 10, 13 and 15 days post-inoculation is displayed. Data correspond to one representative of three independent experiments

To examine the role of Twi1 in plant defence, the wt plants and RNAi Twi1 transgenic lines were inoculated with TSWV. Plants were visually inspected for the development of symptoms, and the percentage of infected plants was recorded at each time point (Fig. 6).

Interestingly, some plants from both RNAi transgenic lines showed symptoms at 6 days post-inoculation (dpi) (10–35%), when all the wt plants were still symptomless. Differences were evident at 8 dpi, when 70 and 90% of transgenic lines 1.1 and 28.3 showed symptoms, respectively, while only 40% of the wt plants displayed disease symptoms. These differences between genotypes continued at 10 dpi. Finally, at 13 dpi the plants from all the genotypes presented symptoms. This result indicates that the defence response appears to be delayed by the absence of the Twi1 enzyme.

RNAi Twi1 transgenic tomato plants infected with TSWV, especially line 28.3, showed much more symptoms in the majority of infection stages compared to the infected wt tomato plants. Particularly, differences in disease severity were statistically significant at 6 and 8 dpi (Additional file 5: Figure S5). In addition, we confirmed that the *Twi1* expression levels in the RNAi transgenic plants infected with TSWV were down-regulated throughout infection (Additional file 6: Figure S6).

### Scopoletin accumulation in the *Twi1*-silenced transgenic tomato plants infected with TSWV

**Fig. 7:**
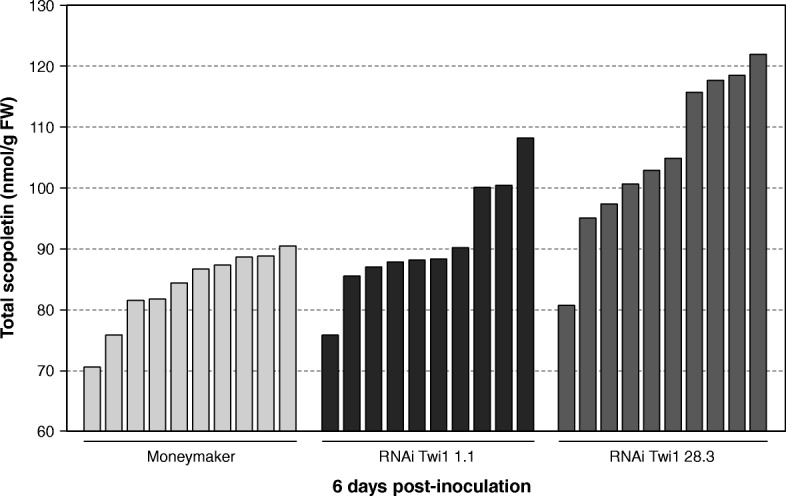
Accumulation of scopoletin in the Moneymaker and RNAi Twi1 transgenic tomato inoculated with Tomato spotted wilt virus (TSWV). Leaf samples were collected at 6 days post-inoculation and were analysed by HPLC-UV. Total scopoletin is the sum of the free and glycosylated forms. Each bar corresponds to one biological replicate (one plant) from one representative experiment. The experiment was repeated 3 times

Accumulation of total scopoletin was studied by HPLC-fluorescence in the RNAi Twi1 transgenic and wt tomato plants infected with TSWV at 6 dpi (Fig. 7), when symptoms started to appear. Regarding total scopoletin (free and conjugated forms), the levels were higher in the majority of the transgenic plants, compared to the wt plants. The accumulation of scopoletin in its free form was not detected in any analysed plant. In order to identify any new relevant compound different from scopoletin and related to the observed TSWV susceptibility in the RNAi Twi1 transgenic plants, we decided to conduct a metabolomic approach.

### Metabolic profiling of *Twi1*-silenced transgenic tomato plants infected with TSWV

**Fig. 8:**
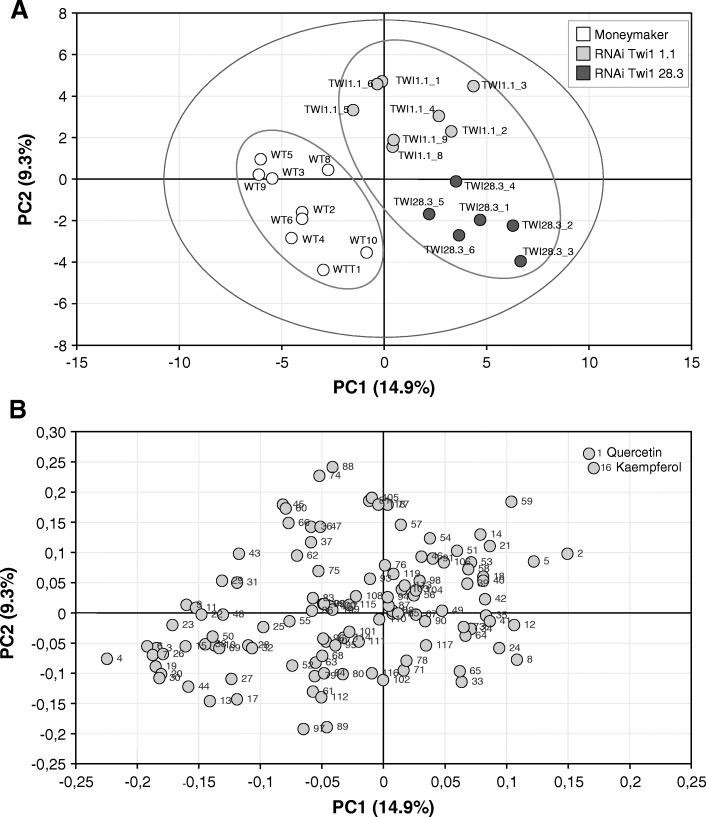
PLS analysis of the metabolites from the Moneymaker and RNAi Twi1 transgenic tomato leaves infected with Tomato spotted wilt virus (TSWV), based on the whole array of the mass spectra within a m/z range from 100 to 1500. **a** The score plot of the hydrolysed organic extracts of the wt Moneymaker (white), RNAi Twi1 1.1 (light grey), and RNAi Twi1 28.3 (dark grey) tomato leaves infected with TSWV at 6 days post-inoculation. **b** The loading plot showing the metabolites that contributed to the separation of the metabolic profiles of the transgenic and parental tomato leaves

Control and RNAi Twi1 samples taken from 6 dpi infected leaves were analysed by UPLC-MS. Then a multivariate data analysis was performed, consisting in a PLS analysis where compound abundance was assigned to the X variable, and genotypes (Moneymaker, RNAi Twi1 1.1 and RNAi Twi1 28.3) were defined as the stepwise Y variable. The PLS analysis (Fig. 8a) showed that the first component (PC1) explained changes in the genotype (wt vs. transgenic plants). The analysis of the PLS loading plot (Fig. 8b) shows the metabolites that contributed to the observed separation. Interestingly, when comparing the metabolome of the RNAi *Twi1*-silenced transgenic tomato plants versus its isogenic parental line, two compounds (1 and 16) were distinctly placed in the positive part of 1-PLS analysis, thus indicating their over-accumulation in the transgenic plants. These compounds were unequivocally identified as the flavonoids quercetin (1) and kaempferol (16).

### Twi1 recombinant protein activity in vitro with flavonoids as substrates

**Fig. 9:**
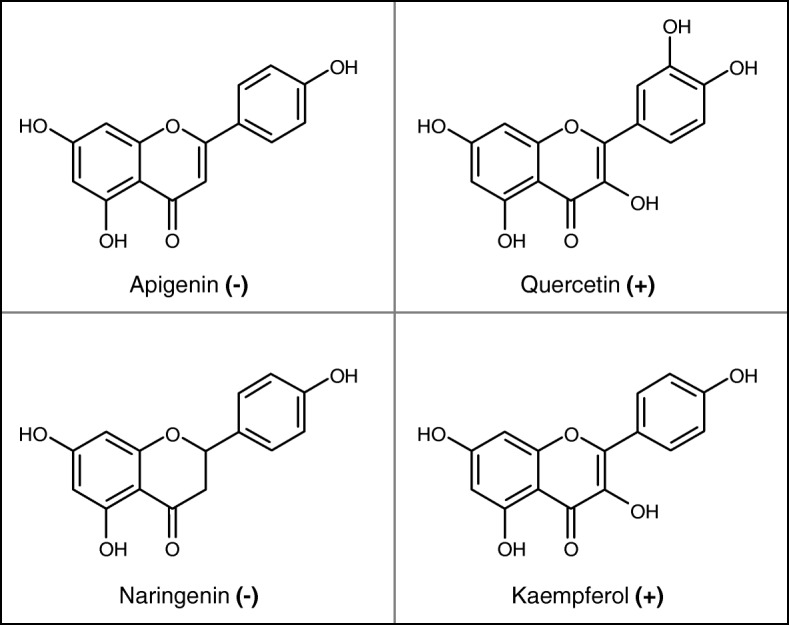
Twi1 in vitro glycosyltransferase activity assay using flavonoids as substrates. The Twi1 purified recombinant protein was tested for glycosyltransferase activity in vitro using UDP-glucose and the following flavonoids as sugar acceptors: apigenin, naringenin, quercetin and kaempferol. Samples were analysed by UPLC-Q-ToF-MS to detect sugar acceptor and glycoconjugate formation. Plus (+): glycoconjugate detected; Minus (−): glycoconjugate not detected

We decided to carry out the in vitro activity assay by testing flavonoids as new substrates for Twi1. The flavonoids tested as sugar acceptors were naringenin, quercetin, kaempferol and apigenin, with UDP-glucose used as the sugar donor. Twi1 showed glucosyltransferase activity towards quercetin and kaempferol (Fig. 9), while no conjugation was observed for naringenin and apigenin, according to our previous PLS analysis results.

### Differential accumulation of flavonoids in the *Twi1*-silenced transgenic tomato plants infected with TSWV

**Fig. 10:**
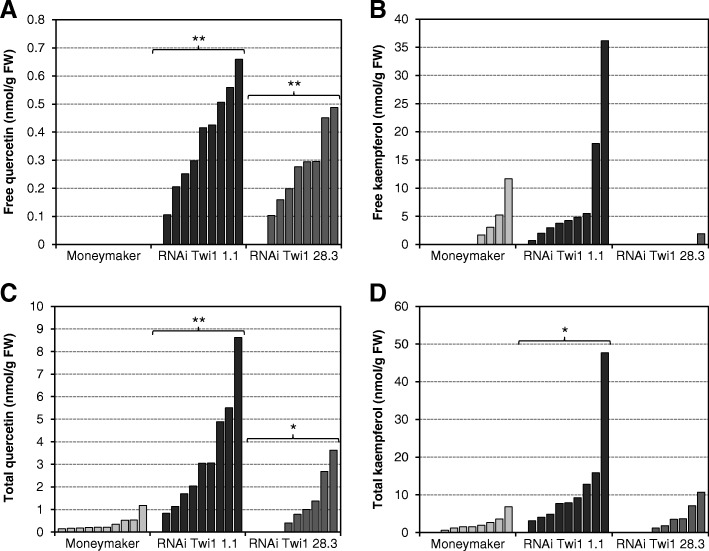
Accumulation of quercetin and kaempferol in the Moneymaker and RNAi Twi1 transgenic tomato inoculated with Tomato spotted wilt virus (TSWV). **a** Free quercetin. **b** Free kaempferol. **c** Total quercetin. **d** Total kaempferol. Leaf samples were collected at 6 days post-inoculation and were analysed by UPLC-Q-ToF-MS. Total is the sum of the free and glycosylated forms. Each bar corresponds to one biological replicate (one plant) from one representative experiment. The experiment was repeated 3 times

A quantitative estimation of free and total quercetin and kaempferol in the RNAi *Twi1-*silenced transgenic plants was made by the UPLC-Q-ToF-MS analysis of the 6 dpi infected leaf samples (Fig. 10). We anticipated that the *Twi1*-silenced plants would present higher free quercetin levels as the silencing of *Twi1* would prevent its conjugation. In agreement with our hypothesis, the free quercetin levels reached values of 0.7 and 0.5 nmol/g FW in transgenic lines 1.1 and 28.3 respectively, while no accumulation was detected in any wt plant (Fig. 10a). The total quercetin levels also significantly increased in the transgenic plants versus the Moneymaker parental. The maximum total quercetin levels in transgenic lines 1.1 and 28.3 were 8.5 and 3.5 nmol/g FW, respectively, and 1.2 nmol/g FW in the wt (Fig. 10c). Between 8 and 10% of the total quercetin accumulated in its free form in the *Twi1*-silenced transgenic lines, while total quercetin corresponded entirely to the conjugated form in the wt plants. The statistical analysis showed that quercetin accumulation in both transgenic lines was significantly greater to that observed in the wt tomato plants.

For kaempferol, the free compound accumulated more in transgenic line 1.1 and reached levels of 36 nmol/g FW, compared to the lesser accumulation of wt that only reached maximum levels of 11 nmol/g FW (Fig. 10b). The free kaempferol accumulation in the transgenic plants from line 28.3 was almost undetectable. Total kaempferol was also higher in line 1.1 compared to the wt, while the levels in line 28.3 remained lower and similar to the wt (Fig. 10d). Total kaempferol accumulation was significant in transgenic line 1.1, and came close to 50 nmol/g FW compared with 10 nmol/g FW in the wt plants. No accumulation of any of both flavonoids was detected in the *mock*-inoculated plants.

## Discussion

Tomato gene *Twi1* is highly induced by wounding, SA and fungal elicitor treatments [41]. The results presented here demonstrate the early induction of *Twi1* in several compatible interactions, such as those produced by the virulent bacteria *P. syringae* in Rio Grande tomato plants carrying the *Pto* gene (Fig.1) and the TSWV virus in Moneymaker tomato plants (Fig. 2), thus suggesting that this gene may be involved in plant defence, like other previously characterized UGTs.

The phylogenetic tree analysis suggested that *Twi1* was likely to encode a scopoletin glucosyltransferase, given its close vicinity to various predicted scopoletin UGTs (Fig. 3). The glycosyltransferase in vitro activity assay of the purified recombinant Twi1 against a variety of phenolic sugar acceptors (Table 1) clearly demonstrated that Twi1: (i) possesses glycosyltransferase activity; (ii) uses UDP-glucose as a sugar donor; (iii), recognises 2,4-DHBA, 2,4,6-THBA, scopoletin, esculetin and umbelliferone as sugar acceptors in vitro and (iv) catalyses the formation of a beta-glucoside bond between glucose and the sugar acceptor (Fig. 4 and Additional file 3: Figure S3 and Additional file 4: Figure S4).

Although the SA treatment strongly induced *Twi1* expression (Additional file 1: Figure S1), no glycosyltransferase activity towards this compound was detected by the in vitro test. In this context, *Twi1* has been described to remain wound-inducible in *NahG* tomato plants, which are unable to accumulate SA, thus indicating that *Twi1* expression is SA-independent [41]. Besides, *TOGT* transcription was stimulated by beta-megaspermin elicitor treatment in *NahG* tobacco plants, which suggests that *TOGT* induction was also SA-independent. In addition, the TOGT in vitro enzymatic assays revealed that SA was a poor substrate, while the coumarins scopoletin and esculetin proved to be the best sugar acceptors [26], which is in accordance with the phylogenetic proximity between TOGT and Twi1 (Fig. 3).

Similarly to other GTs, Twi1 seemed fairly specific to the sugar donor while recognising various substrates in vitro [26, 38, 42–48]. Accumulation of coumarins has been reported to occur in plants in response to wounding and infections with different pathogens [9, 49–59]. They also display antimicrobial and antioxidant activities [32, 60–62]. Moreover, treatment with esculetin completely abolish both viral-induced cell death and production of Coccolithovirus EhV in the alga *Emiliania huxleyi* [63].

Among the compounds identified as Twi1 in vitro substrates, no role had been assigned to 2,4-DHBA and 2,4,6-DHBA in plants, in contrast with other SA derivatives, such as 2,3-DHBA and 2,5-DHBA [64–67]. Considering this, we focused our study on the coumarins as putative Twi1 substrates *in planta*.

In many cases, glycosyltransferases have been reported to be rapidly induced by their own substrates [18, 26, 42]. According to this, treatments by stem-feeding with scopoletin, esculetin and umbelliferone resulted in a clear induction of *Twi1* in the Moneymaker tomato plants (Fig. 5). In addition, *Twi1*-silenced transgenic Moneymaker tomato plants were generated and analysed for *Twi1* induction after the same treatments. Two obtained homozygous lines, Twi1 RNAi 1.1 and 28.3, showed a sharp drop in the *Twi1* transcripts, confirming the efficacy of the gene silencing strategy.

In order to study the role of Twi1 in plant defence, the RNAi transgenic tomato lines were inoculated with TSWV. Plants from both RNAi transgenic lines showed symptoms at 6 dpi, when all the wt plants were still symptomless (Fig. 6). Differences were evident at 8 dpi and 10 dpi, while at 13 dpi the plants from all the genotypes presented symptoms. This is in agreement with the disease severity, reaching RNAi Twi1 transgenic lines higher percentages, especially line 28.3, that showed much more symptoms in the majority of infection stages compared to the infected wt tomato plants (Additional file 5: Figure S5). We have shown that a reduced *Twi1* expression diminishes resistance to viral infection in tomato, suggesting a role of this glycosyltransferase in plant defence. Similar results have been reported in various plant-pathogen interactions. In Arabidopsis, the expression of two glycosyltransferases, UGT73B3 and UGT73B5, was necessary for resistance to *P. syringae* [28]. Besides, *ugt* mutants showed a reduced resistance phenotype [30]. The heterologous expression of SsGT1 glycosyltransferase in flax (*Linum usitatissimum*) increased resistance to *Fusarium* infection [68], and VIGS suppression of the *CaUGT1* gene in hot pepper resulted in a delayed HR phenotype against TMV and in greater viral coat protein accumulation [29].

Metabolites from RNAi Twi1 transgenic and wt tomato plants infected with TSWV at 6 dpi were analysed by HPLC-fluorescence in order to detect coumarin accumulation. Among them, only scopoletin showed to accumulate in the infected tomato plants. Levels of total scopoletin were higher in the majority of the transgenic plants (Fig. 7), compared to wt, while accumulation of scopoletin in its free form was not detected. This accumulation correlates with the reported levels of scopolin (scopoletin glucoside) measured in the tobacco plants infected with TMV [27].

Our results contrast with the expected phenotype for the RNAi Twi1 transgenic tomato plants, as we speculated that GT silencing would lead to a decrease in the conjugated form of the substrate, and consequently to an increase in the free form. But similar controversial results have been previously reported when overexpressing or silencing different plant GTs. For instance, *Arabidopsis* transgenic plants overexpressing *AtSGT1*, which encodes an SA glycosyltransferase, showed lower levels of free and conjugated SA, compared to wt plants [69]. A drop in the amount of scopolin and scopoletin has been reported in *TOGT*-silenced transgenic tobacco plants after infection with TMV, compared to wt plants [27]. Moreover, *TOGT*-overexpressing transgenic plants accumulated twofold more scopolin and scopoletin after inoculation with TMV versus wt tobacco [32]. To better clarify this, we carried on a metabolomic analysis in order to identify any new relevant compound related to the observed TSWV susceptibility transgenic plants.

Control and RNAi Twi1 samples taken from the 6 dpi infected leaves were analysed by UPLC-MS, and a multivariate data analysis was performed. The PLS analysis revealed that the metabolic content markedly contributed to the separation of samples in both groups (wt vs. transgenic plants) (Fig. 8). Among metabolites, two flavonoids, quercetin and kaempferol, showed to over-accumulate in the transgenic plants, meaning they were probably involved in the role of Twi1 in plant defence.

We performed the activity assay in order to test flavonoids as new in vitro substrates for Twi1. Among them, Twi1 showed glucosyltransferase activity towards quercetin and kaempferol (Fig. 9), indicating that these two flavonoids may be Twi1 in vivo substrates as well. The lack of glycosyltransferase activity of Twi1 against narigenin and apigenin could be related with the different chemical structure of these flavonoids in comparison to quercetin and kaempferol, OH-3 being probably involved in the substrate specificity. Further modelling studies could help better understand this point.

To date, several flavonoid GTs have been identified and characterised in different plant species [11, 37, 70]. Similar to our results, in strawberry UGT71A34a and UGT71A34b presented in vitro activity towards both coumarins (scopoletin and esculetin) and flavonoids (quercetin and kaempferol) [34]. In tobacco, the scopoletin glucosyltransferase SGTase, which prefers hydroxycoumarins, also exhibits significant activity with flavonoids [43] when characterising. These authors postulated that SGTase would work as a multiple phenylpropanoid glucosylation enzyme by regulating the storage site of these compounds for further utilisation, or to avoid their toxic effect. We similarly propose that Twi1 could act as a multiple phenolic glucosylation enzyme in tomato plants.

Flavonoids are synthesised by a wide variety of plants and perform many diverse biological functions [71–73]. The role of flavonoids in plant defence has been reviewed in-depth [74]. Several in vitro studies have shown that flavonoids display antimicrobial activity against bacteria and fungi [75, 76], and pre-incubation with sulphated and methylated derivatives of quercetin decreases TMV infectivity by 40–50% [77]. Accumulation of quercetin and kaempferol has been reported in plants in response to pathogens, mostly fungal infections. Not only the free form, but also glycosylated flavonoids have been shown to also possess excellent antioxidant properties [78]. Rutin, a quercetin-3-*O*-rutinoside, rapidly accumulates in tomato leaves upon *P. syringae* inoculation [79], and in potato plants inoculated with *Verticillium dahliae* [13]. This and other previous studies have shown the inhibitory effects of rutin and quercetin on the growth of *V. dahliae* in vitro and in vivo [12]. A higher accumulation of these phenolic antioxidant metabolites occurs in buckwheat varieties less susceptible to the fungus *Aspergillus flavus* [80], and in barley genotypes resistant to *Gibberella zeae* [81].

The main role attributed to flavonoids in plant defence is due to their antioxidant properties [15], which allow them to quench ROS and reduce their production generated by the pathogen and the plant itself during infection. Other possible roles of flavonoids in plant defence have been described, such as cell wall reinforcement to restrict pathogen access to nutrients, and the inhibition of the plant cell wall degrading enzymes produced by fungi [74, 82].

Levels of quercetin and kaempferol in the RNAi *Twi1-*silenced transgenic plants were quantified at 6 dpi infected leaves (Fig. 10). We detected that free and total levels of quercetin were statistically higher in both RNAi Twi1 transgenic plants, being the observed differences more notable in the 1.1 transgenic line. Regarding kaempferol levels, the same tendency was observed, detecting statistically differences only in the total levels of the RNAi Twi1 1.1 transgenic plants. The higher activity obtained for the quercetin could be due to a higher binding affinity or efficacy of Twi1 for this flavonoid with respect to the kaempferol. To our knowledge, this is the first time that the accumulation of quercetin and kaempferol is reported in response to viral infection in plants. Considering*Twi1*-silenced transgenic lines were hypersusceptible to TSWV compared to the wt plants, their higher content in flavonoids contrasts with previous plant-pathogen interaction studies, where higher levels of these compounds have normally been associated with resistance [80, 81].

It should be noted that, even when *Twi1* expression was nearly suppressed in the transgenic lines, the quercetin and kaempferol conjugated forms were still produced, thus suggesting that other GTs may exist or are induced due to *Twi1* silencing, which would thus explain their conjugation. This agrees with Gachon and collaborators [32], who suggested that scopoletin is conjugated by various enzymes in tobacco. The biological relevance of plants having more than one UGT acting on the same substrate has been recently discussed [83], and is justified as the attempt of keeping the fine-tuning of metabolic homeostasis and avoiding the possible toxic effects of the aglycone. We may consider the possibility that other GTs would glycosylate quercetin and kaempferol in a different hydroxyl position, or with a different sugar molecule. However, since the precise structure of the Twi1 product has not been elucidated, further studies would be necessary to address this possibility. The putative change in conjugation would disturb the regulatory network of these compounds in the cell by, for instance, preventing their degradation, so they would accumulate in larger amounts in transgenic plants compared to the wt. The biological properties of the glycosylated compounds, such as their antioxidant activity, would be altered due to a modification in the glycosylation pattern. Both effects could cooperate for the observed susceptibility of the RNAi Twi1 transgenic tomato plants to TSWV. The further characterisation of the quercetin and kaempferol glycosides produced in the RNAi Twi1 transgenic plants would be interesting to better understand the viral susceptibility.

## Conclusions

Metabolomics, in conjunction with biochemical and in vivo analyses, have revealed that *Twi1*, formerly described as a wound- and SA-induced gene encoding a putative UGT [41], is in fact a tomato glycosyltransferase involved in the metabolism of not only scopoletin, but also of quercetin and kaempferol, and it is involved in plant defence against virus. The study of various GTs has revealed that some are more promiscuous than expected from the literature [48]. Therefore, the predicted function for several UGTs and the substrate specificity of some already characterised UGTs could be reconsidered. The new role uncovered for Twi1 strongly supports the notion that the functions and specificities of UGTs are perhaps not accurately determined based exclusively on their amino acid sequence alignments [11, 84]. The coupling of metabolomics with experimental analyses should be considered as a much more efficient approach for UGT characterisation.

## Methods

### Plant material

Tomato *(Solanum lycopersicum)* and *Nicotiana benthamiana* (lab strain) plants were used in this study. Two different cultivars of tomato plants were used: ‘Rio Grande’ containing the *Pto* resistance gene (gently provided by Dr. Selena Giménez, Centro Nacional de Biotecnología, Madrid, Spain), and ‘Moneymaker’ (a gift from Prof. Jonathan Jones, The Sainsbury Laboratory, Norwich, UK). Plants were grown under standard greenhouse conditions (temperature from 20 to 25 °C, 16-h day/8-h night photoperiod, relative humidity from 50 to 70%). Pots were subirrigated once a day with nutrient Hoagland solution [85].

### Chemical treatments

The tomato plant treatments were carried out by stem-feeding [86]. Tomato 4-week old plants were excised with a scalpel just above cotyledons, and stems were immersed in the different compounds. After 30 min, all the stems were transferred to water and leaf tissue was collected at the indicated times. For the SA treatments, 2 mM SA were used, and samples were taken at 0, 0.5, 1, 8 and 24 h post-treatment. A sample was collected before starting to perform the experiment. For the scopoletin, umbelliferone or esculetin treatments, tomato plants were also stem-fed for 30 min, but this time stems were immersed in 0.5 mM of the corresponding phenolic compounds, and samples were collected at 0, 1, 6 and 24 h post-treatment. All the treatments were performed in a growth chamber at a constant temperature of 24 °C and a photoperiod of 16 h of light (300 μmol/m^2^/s) and 8 h of darkness. Both the third and fourth leaves from explants were harvested at the indicated times.

### Bacterial growth conditions, inoculum preparation and inoculation

The bacterial strains herein used were *Pseudomonas syringae* pv. *tomato* DC3000 (*Pst* DC3000 *AvrPto*) and *Pst* DC3000, which contains deletions in genes *AvrPto* and *AvrPtoB* (*Pst* DC3000 ∆*AvrPto*) [87]. The inoculum preparation and infection was carried out according to López-Gresa and collaborators [88]. The third and fourth leaves, from bottom to top, were harvested at 0, 10, 18, 24, 36 and 48 h post-inoculation. Three biological replicates were analysed for each time and tomato-bacteria interaction.

### TSWV inoculation

Tomato plants were inoculated with TSWV according to Soler and collaborators [89]. Briefly, 1 gram of the TSWV-infected leaves was homogenised in 20 mL of phosphate buffer (3 mM monosodium phosphate, 75 mM disodium phosphate, pH 7.4) containing 0.15 M NaCl, 1% polyvinylpolypyrrolidone, 0.02% mercaptoethanol, 1% carborundum and 1% active carbon. The 4-week old plants were inoculated on the uppermost leaflet from the third and fourth leaves (leaf position numbered from the base to the apex) using 50–100 μL of the viral extract per leaflet. Plants were dusted with carborundum, and were then inoculated by rubbing with a cotton swab soaked in virus suspension or were *mock*-inoculated with buffer only. One week later, the fifth and sixth leaves were inoculated. The fifth and sixth leaves were collected for all the analytical measurements at the indicated time points of infection. Plants were inspected visually for symptom evaluation, and disease severity was scored at the indicated time points. Samples corresponding to 3 replicates for the time course RNA analysis were collected and frozen at − 80 °C.

To determine the metabolite content, new sets of experiments were performed using 10 tomato plants of each genotype (Moneymaker, RNAi Twi1 1.1 and RNAi Twi1 28.3) and sampling at 6 dpi.

### RNA extraction and quantitative RT-PCR analysis

The total RNA of the tomato tissues was isolated using TRIzol reagent (Invitrogen) following the manufacturer’s protocol. A quantitative RT-PCR analysis was performed as previously described in the work by Campos and collaborators [90]. A house-keeping gene transcript, *Elongation Factor 1 alpha* (*eEF1α*), was used as an endogenous reference. The PCR primers used to amplify *Twi1* were 5′-GGATGCGAAGAGCTATGGAG-3′ as the forward primer and 5′-CGGACCAATAGCCCAATTTT-3′ as the reverse primer. For *eEF1α* amplification*,* 5′-CCACCTCGAGATCCTAATGG-3′ and 5′-ACCCTCACGTATGCTTCCAG-3′ were used as the forward and the reverse primer, respectively.

### Vector construction

The full-length cDNA (1412 bp) of *Tomato wound-induced* gene (*Twi1*) [41] was amplified by RT-PCR from the Moneymaker tomato leaves infected with the bacterial pathogen *Pseudomonas syringae* pv. *tomato* using 5′-ATGGGTCAGCTACATTTTTTC-3′ as the forward primer and 5′-TTAACGATATGAAGTTATGTC-3′ as the reverse primer. The resulting PCR product was cloned into the pCR8/GW/TOPO entry vector (Invitrogen), following the manufacturer’s protocol, and was sequenced. Then *Twi1* was subcloned in the pGWB8 Gateway binary vector [91]. In order to generate the *Twi1*-silenced transgenic tomato plants, the method described by Helliwell and Waterhouse was followed [92]. Briefly, a selected 341 bp sequence of *Twi1* was amplified from the full-length cDNA clone using the forward primer 5′-GGCTCGAGTCTAGAGAAATCAAGTTCCATTGTTTAT-3′, which introduced restriction sites *Xho*I and *Xba*I, and the reverse primer 5′-CCGAATTCGGATCCACTTCTCATTGAAAAAC-3′, which added restriction sites *Bam*HI and *Eco*RI. The PCR product was first cloned in the pGEM T Easy vector (Promega) and sequenced. After digestion with the appropriate restriction enzymes and purification, the two *Twi1* fragments were subcloned into the pHANNIBAL vector in both the sense and antisense orientations. Finally, the constructs made in pHANNIBAL were subcloned as a *Not*I flanked fragment into binary vector pART27 to produce highly effective intron-containing “hairpin” RNA silencing constructs. This vector carries the *neomycin phosphotransferase* gene (*NPT II*) as a transgenic selectable marker.

### *N. benthamiana* agroinfiltration and tomato transformation

The pGWB8-*Twi1* construction and the pGWB8 empty vector were transformed into the *Agrobacterium tumefaciens* C58 strain, while the pART27-*Twi1* construction was transformed into *A. tumefaciens* LBA4404. The leaves of the 4-week-old *N. benthamiana* plants were infiltrated with the *A. tumefaciens* C58 carrying pGWB8-*Twi1* or the empty vector, and with the C58 strain carrying the p19 plasmid (1:1), which encodes silencing suppressing protein p19 [93].

The transformed LBA4404 *A. tumefaciens* carrying pART27-*Twi1* was co-cultured with the tomato Moneymaker cotyledons to generate the RNAi *Twi1*-silenced transgenic tomato plants (RNAi Twi1). The explant preparation, selection and regeneration methods followed those published by Ellul and co-workers [94]. The tomato transformants were selected in kanamycin-containing medium and propagated in soil. The Moneymaker tomato wild-type plants regenerated in vitro from cotyledons under the same conditions as the transgenic lines were used as controls in subsequent analyses. The transgenic plants generated in this study have been identified and characterised in our laboratory and are to be used exclusively for research purposes.

### Metabolite extraction procedure

Extraction of the methanol-soluble compounds from tomato leaves was performed according to López-Gresa and collaborators [79]. Tissue (0.5 g fresh weight) was ground to powder in a mortar using liquid nitrogen, and then homogenized in 1.5 mL 100% methanol. The extracts were sonicated for 10 min and centrifuged for 15 min at 10000 x *g* to remove cellular debris. The supernatant corresponding to each sample was divided in two equal portions and dried at 40 °C with a flow of nitrogen. One half of the dried residue was resuspended in 900 μL of 50 mM sodium acetate (pH 4.5) and 100 μL of water containing 10 U of almond beta-glycosidase (EC 3.2.1.21) (14.3 U/mg, Fluka) to analyse total (free + conjugated) forms. The other half was resuspended in 900 μL of 50 mM sodium acetate (pH 4.5) and 100 μL of water to analyze free form. The reactions were incubated overnight at 37 °C and stopped by adding 75 μL of 70% perchloric acid to the incubation mixtures (5% (v/v) final concentration). After centrifugation at 14000 x *g* for 15 min to remove polymers, the supernatants were extracted with 2.5 mL of cyclopentane/ethyl acetate (1:1, v/v). The organic upper phase was collected and dried at 40 °C under a flow of nitrogen. The residue was resuspended in 100 μL of methanol and filtered through 13-mm nylon 0.45 μm Minispike filters (Waters) prior to analysis.

### HPLC analysis

The HPLC analysis was performed following Yalpani and coworkers [95], slightly modified by Campos et al. [96]*,* in which fluorescence detection was used. Compounds (SA, 2,4-dihydroxybenzoic acid (DHBA), scopoletin, esculetin, and umbelliferone) were detected by a 470 Waters fluorescence detector (λ excitation 313 nm; λ emission 405 nm), and quantified with the Waters Empower software using commercial compounds as a standard. Data were corrected for losses in the extraction procedure, and the recovery of metabolites ranged between 50 and 80%.

For the in vitro assay test, a 20 μL aliquot from the final 200 μL volume reaction was injected into HPLC-UV to detect SA, 2,4-DHBA, scopoletin, esculetin and umbelliferone. Commercial standards were used to quantify the results.

### UPLC-PDA-micromass Q-ToF analysis

A 5 μL aliquot from the final 100 μL sample extraction was analysed by UPLC-MS by an ACQUITY UPLC-PDA system coupled to a Q-ToF Micromass spectrometer (Waters) according to Campos and co-workers [96]. All the data were acquired with the Masslynx NT4.1 software (Waters Corp. Mildford, MA, USA). For the untargeted analysis of the hydrolysed polar and semi-polar compounds, a metabolomic study of the total forms was performed using the negative ESI-MS spectra.

For the in vitro assay test, a 5 μL aliquot from the final 200 μL volume reaction was injected into UPLC-PDA-MS to detect and quantify with standards the phenylpropanoids cinnamic acid, *p*-coumaric acid, caffeic acid, ferulic acid, *o*-coumaric acid and chlorogenic acid, the flavonoids quercetin, kaempferol, naringenin and apigenin, and the simple phenolics benzoic acid, 4-hydroxybenzoic acid and 2,4,6-trihydroxybenzoic acid (THBA).

### Twi1 recombinant protein purification

Four grams of frozen *N. benthamiana* leaves infiltrated with recombinant *A. tumefaciens* C58, carrying either pGWB8-*Twi1* or the pGWB8 empty vector (control), were ground to a fine dust in liquid nitrogen and resuspended in 8 mL of extraction buffer (20 mM sodium phosphate, 0.5 M NaCl, 40 mM imidazole, pH 7.4, containing 80 μL of PMSF 1 mM and 16 μL 2-mercaptoethanol). The plant material was homogenised, filtered through Miracloth (Calbiochem), and tissue debris was removed by two successive centrifugations at 10,000 x *g* and 4 °C for 15 min. The sample was then filtered through 4-mm and 0.45-μm pore diameter nylon membranes (Waters). Protein samples were subjected to FPLC (Fast Protein Liquid Chromatography) with a nickel-loaded HisTrap HP 1 mL column (GE Healthcare Life Sciences). The column was washed with binding buffer (20 mM sodium phosphate, 0.5 M NaCl, 40 mM imidazole, pH 7.4), and the retained proteins were eluted with a 40–500 mM linear imidazole gradient in the same buffer at a flow rate of 1 mL/min by measuring A_280_ at the column outlet and collecting 500 μL fractions by a RediFrac-1 automatic collector (Amersham Pharmacia Biotech).

Fractions were pooled in threes and analysed by SDS-PAGE following the method described by Conejero and Semancik [97]. Proteins were stained with Coomassie Brilliant Blue R-250. The recombinant Twi1 protein appeared only in the leaf tissues infiltrated with the pGWB8-*Twi1* construction. The fractions containing the recombinant Twi1 protein were desalted using a PD-10 column, eluted with Tris buffer (Tris-HCl 50 mM, pH 7.5), and assayed for activity in vitro. The equivalent fractions from the control sample were also tested.

### In vitro assay of recombinant Twi1 activity

The reaction mixture used to perform the standard assay for glycosyltransferase activity contained: 200 μL of the purified protein in Tris buffer (Tris-HCl 50 mM, pH 7.5), 0.1 mM of the final concentration of the sugar acceptor and 2 mM UDP-glucose (Fluka) or UDP-xylose (CarboSource Services, Complex Carbohydrate Research Centre, University of Georgia, USA) in a 206,5 μL final volume reaction. All the compounds used for Twi1 glycosyltransferase activity test were purchased from Sigma. Reactions were incubated overnight at 37 °C and stopped by adding one volume of 100% methanol. Then samples were filtered through a 4-mm nylon membrane (0.45 μm pore, Waters) and were analysed by high performance liquid chromatography (HPLC, Waters) according to the Yalpani [98] and Bellés [64] indications, or by a Q-ToF-MS analysis.

To detect the conjugated products of SA, GA, 2,4-DHBA, scopoletin, esculetin and umbelliferone, HPLC-fluorescence was employed as described before. The conjugated forms of the other simple phenolics and of the phenylpropanoids were detected by Q-ToF-PDA-MS, as reported above.

### Bioinformatics and statistical analyses

The symptomatology of each plant, which was monitored at the indicated time points and scored according to symptom severity, was statistically analysed by a Kruskal-Wallis test. Different letters indicate significant differences (*p*-value < 0.05) between the RNAi Twi1 transgenic and wt infected plants.

The “Infectivity Index” consists of the total number of days that each plant presents symptoms [90]. The data from a representative experiment of three independent assays were used to perform the statistical analysis by the Mann-Whitney nonparametric test. A *p*-value of < 0.05 was considered statistically significant. The IBM SPSS v.19 package was used for all the statistical analyses.

For the untargeted analysis of the hydrolysed polar and semi-polar profiles, the UPLC-MS data were processed with XCMS online resources (https://xcmsonline.scripps.edu) with the appropriate script for the alignment of chromatograms and the quantification of each MS feature [99]. The resulting dataset was submitted to a Partial Least Square (PLS) study by the SIMCA-P software (v. 11.0, Umetrics, Umeå, Sweden) using unit variance (UV) scaling.

The phylogenetic tree analysis was generated with the MegAlign software bundled in the DNASTAR Lasergene package (DNASTAR Inc., Madison, WI, USA). The glycosyltransferases considered in the alignment were: IS5a (GB: AAB36653), Togt1 (GB: AAK28303), Togt2 (GB: AAK28304), IS10a (GB: AAB36652), SA-GTase (GB: AAF61647) and JIGT (GB: BAA19155) from *Nicotiana tabacum*; Scopoletin GT (NCBI: XP_015062099) from *Solanum pennellii*; Sgt1 (GB: AAB48444) and Scopoletin GT (NCBI: XP_006346388) from *Solanum tuberosum*; Scopoletin GT (NCBI: XP_016539537) from *Capsicum annuum*, UGT73B3 (NCBI: NP_567953) and UGT73B5 (NCBI: NP_179150) from *Arabidopsis thaliana*, as well as Twi1 (GB: CAA59450) and GAGT (GB: CAI62049) from *Solanum lycopersicum*.

## Supplementary information

**Additional file 1: Figure S1.** Gene expression of *Twi1* in tomato leaves upon water or salicylic acid treatment.

**Additional file 2: Figure S2.** Purification of the Twi1 recombinant protein.

**Additional file 3: Figure S3.** Twi1 enzyme activity towards umbelliferone, 2,4-DHBA and sculetin.

**Additional file 4: Figure S4.** Twi1 enzyme activity towards 2,4,6-DHBA.

**Additional file 5: Figure S5.** Disease severity of the transgenic and parental plants to TSWV virus.

**Additional file 6: Figure S6.** *Twi1* gene expression transgenic tomato plants upon TSWV infection.

## Data Availability

Material will be available upon request to authors after MTA signature.

## Abbreviations

2,4,6-THBA: 2,4,6-trihydroxybenzoic acid
2,4-DHBA: 2,4-dihydroxybenzoic acid
BLAST: Basic local alignment search tool
CAZy: Carbohydrate active enzymes
CEVd: Citrus exocortis viroid
GA: Gentisic acid
GT: Glycosyltransferase
HPLC: High performance liquid chromatography
MS: Mass spectrometry
PCR: Polymerase chain reaction
PLS: Partial least squares
PSPG: Plant secondary product glycosyltransferase
*Pst*: *Pseudomonas syringae* pv. *tomato*
Q-ToF: Quadrupole-time of flight
RNAi: Interfering RNA
SA: Salicylic acid
TMV: Tobacco mosaic virus
TOGT: Tobacco glucosyltransferase
ToMV: Tomato mosaic virus
TSWV: Tomato spotted wilt virus
UGT: Uridine diphosphate sugar-dependent glycosyltransferase
UPLC: Ultra performance liquid chromatography
wt: Wild type
