# Supplementary material for: Tomato glycosyltransferase Twi1 plays a role in flavonoid glycosylation and defence against virus
Source: BMC Plant Biol. 2019 Oct 26;19:450. doi: 10.1186/s12870-019-2063-9 (PMC6815406; doi:10.1186/s12870-019-2063-9)
Supplement: Supplementary file 1 — Additional file 1: Figure S1. Gene expression of Twi1 in tomato leaves upon water or salicylic acid treatment. [file 12870_2019_2063_MOESM1_ESM.pptx]

## Slide 1
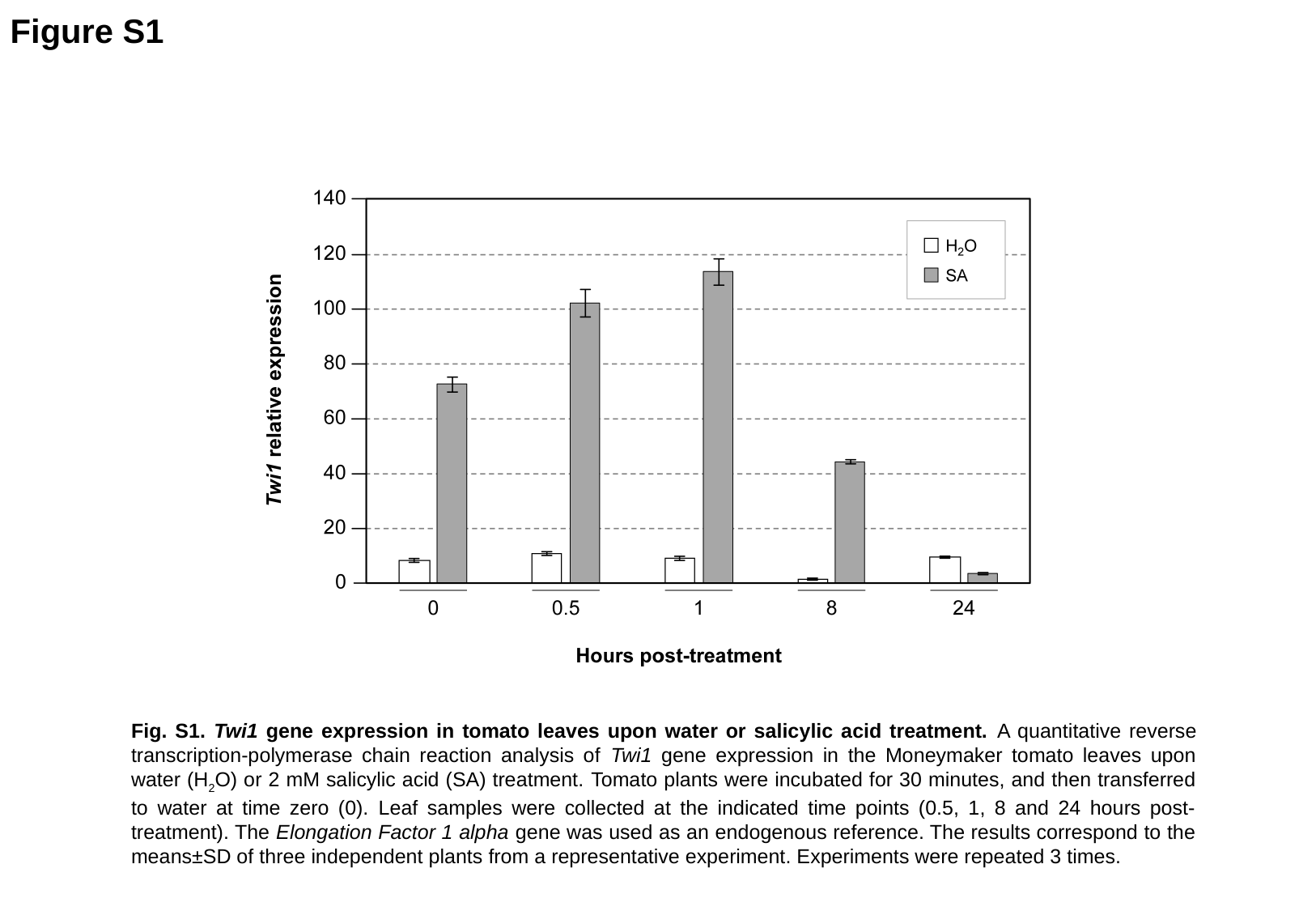

Figure S1
Fig. S1. Twi1 gene expression in tomato leaves upon water or salicylic acid treatment. A quantitative reverse transcription-polymerase chain reaction analysis of Twi1 gene expression in the Moneymaker tomato leaves upon water (H2O) or 2 mM salicylic acid (SA) treatment. Tomato plants were incubated for 30 minutes, and then transferred to water at time zero (0). Leaf samples were collected at the indicated time points (0.5, 1, 8 and 24 hours post-treatment). The Elongation Factor 1 alpha gene was used as an endogenous reference. The results correspond to the means±SD of three independent plants from a representative experiment. Experiments were repeated 3 times.
