# Supplementary material for: Tomato glycosyltransferase Twi1 plays a role in flavonoid glycosylation and defence against virus
Source: BMC Plant Biol. 2019 Oct 26;19:450. doi: 10.1186/s12870-019-2063-9 (PMC6815406; doi:10.1186/s12870-019-2063-9)
Supplement: Supplementary file 2 — Additional file 2: Figure S2. Purification of the Twi1 recombinant protein. [file 12870_2019_2063_MOESM2_ESM.pptx]

## Slide 1
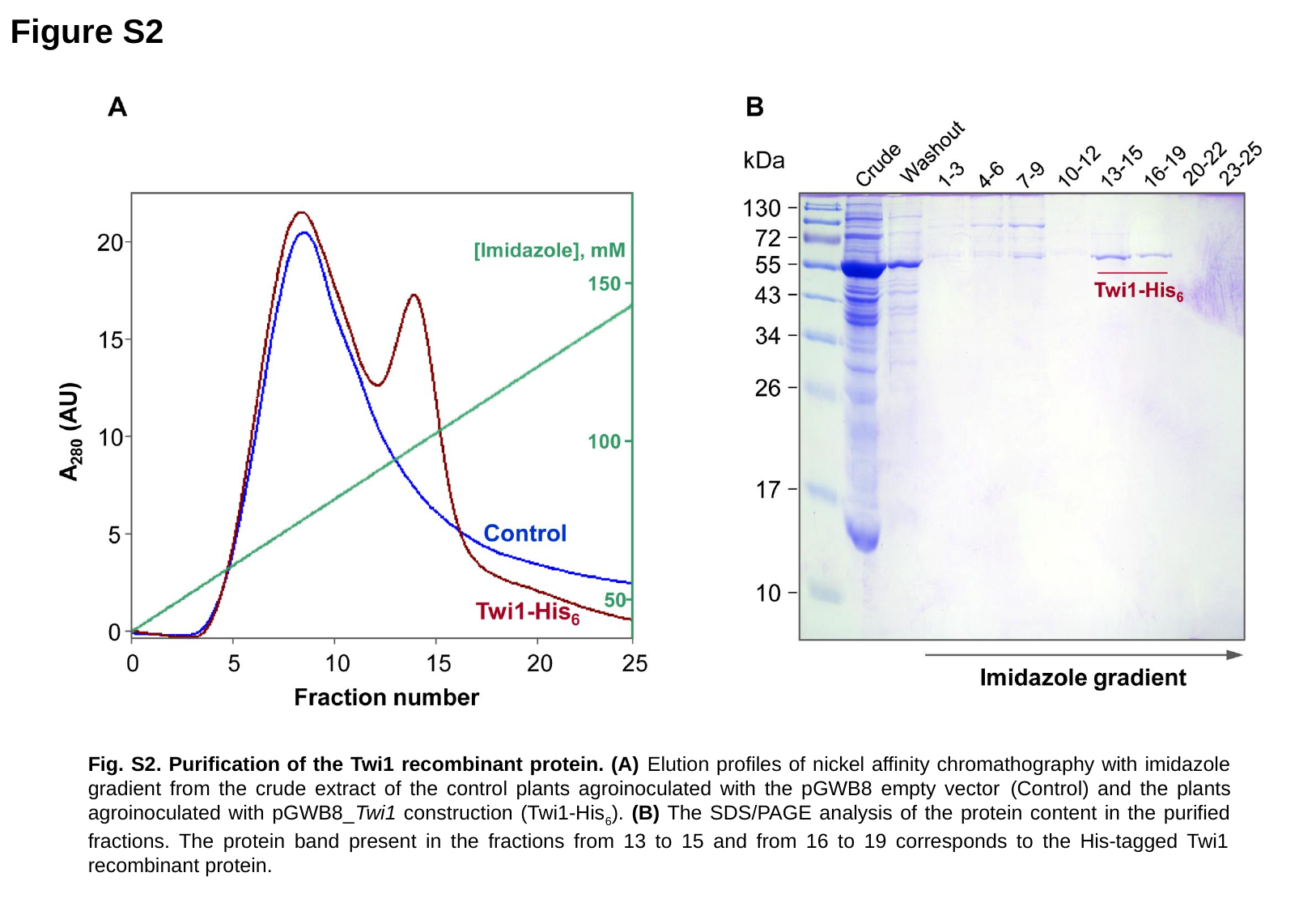

Figure S2
Fig. S2. Purification of the Twi1 recombinant protein. (A) Elution profiles of nickel affinity chromathography with imidazole gradient from the crude extract of the control plants agroinoculated with the pGWB8 empty vector (Control) and the plants agroinoculated with pGWB8_Twi1 construction (Twi1-His6). (B) The SDS/PAGE analysis of the protein content in the purified fractions. The protein band present in the fractions from 13 to 15 and from 16 to 19 corresponds to the His-tagged Twi1 recombinant protein.
