# Supplementary material for: Tomato glycosyltransferase Twi1 plays a role in flavonoid glycosylation and defence against virus
Source: BMC Plant Biol. 2019 Oct 26;19:450. doi: 10.1186/s12870-019-2063-9 (PMC6815406; doi:10.1186/s12870-019-2063-9)
Supplement: Supplementary file 3 — Additional file 3: Figure S3. Twi1 enzyme activity towards umbelliferone, 2,4-DHBA and sculetin. [file 12870_2019_2063_MOESM3_ESM.pptx]

## Slide 1
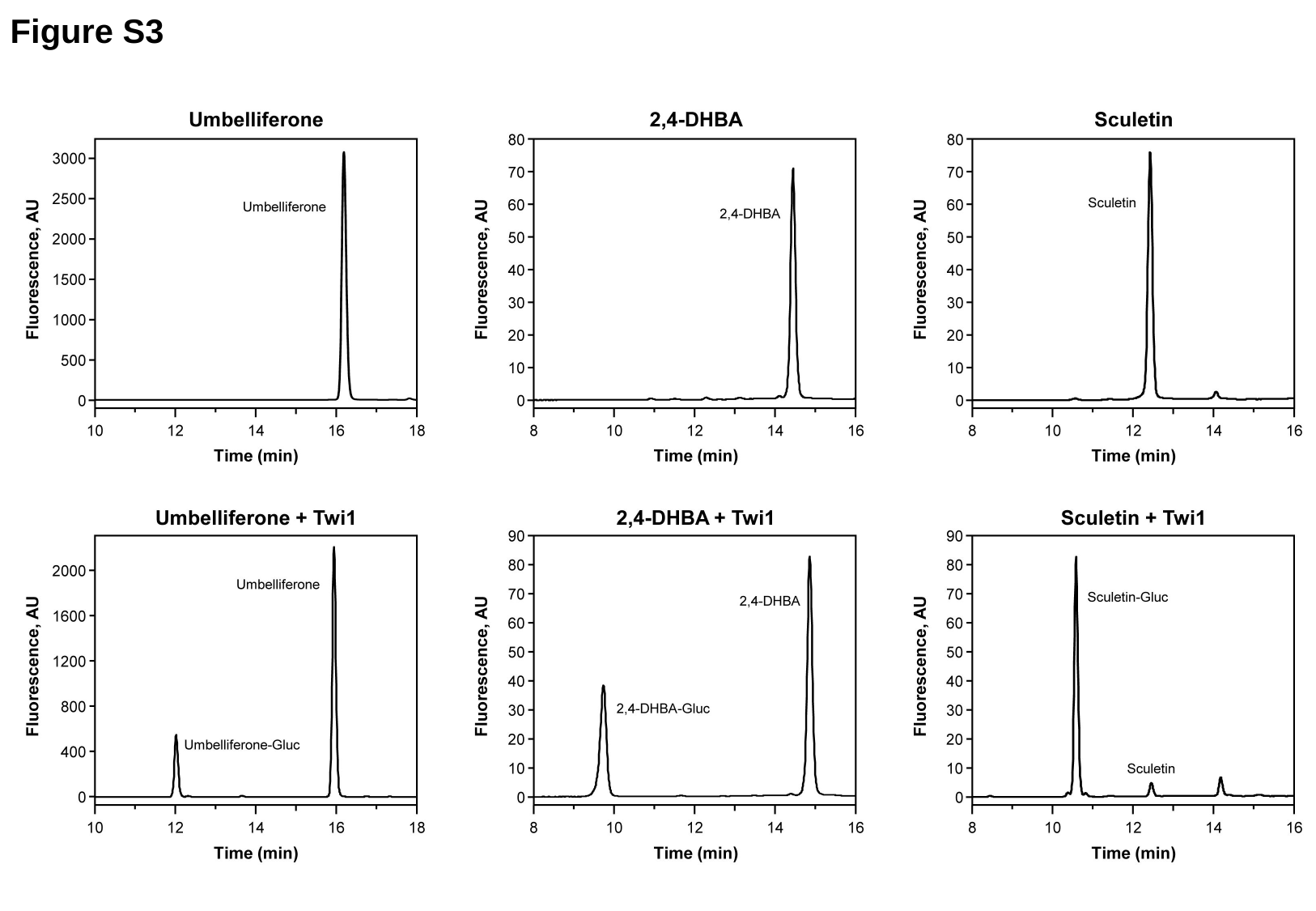

Figure S3

## Slide 2
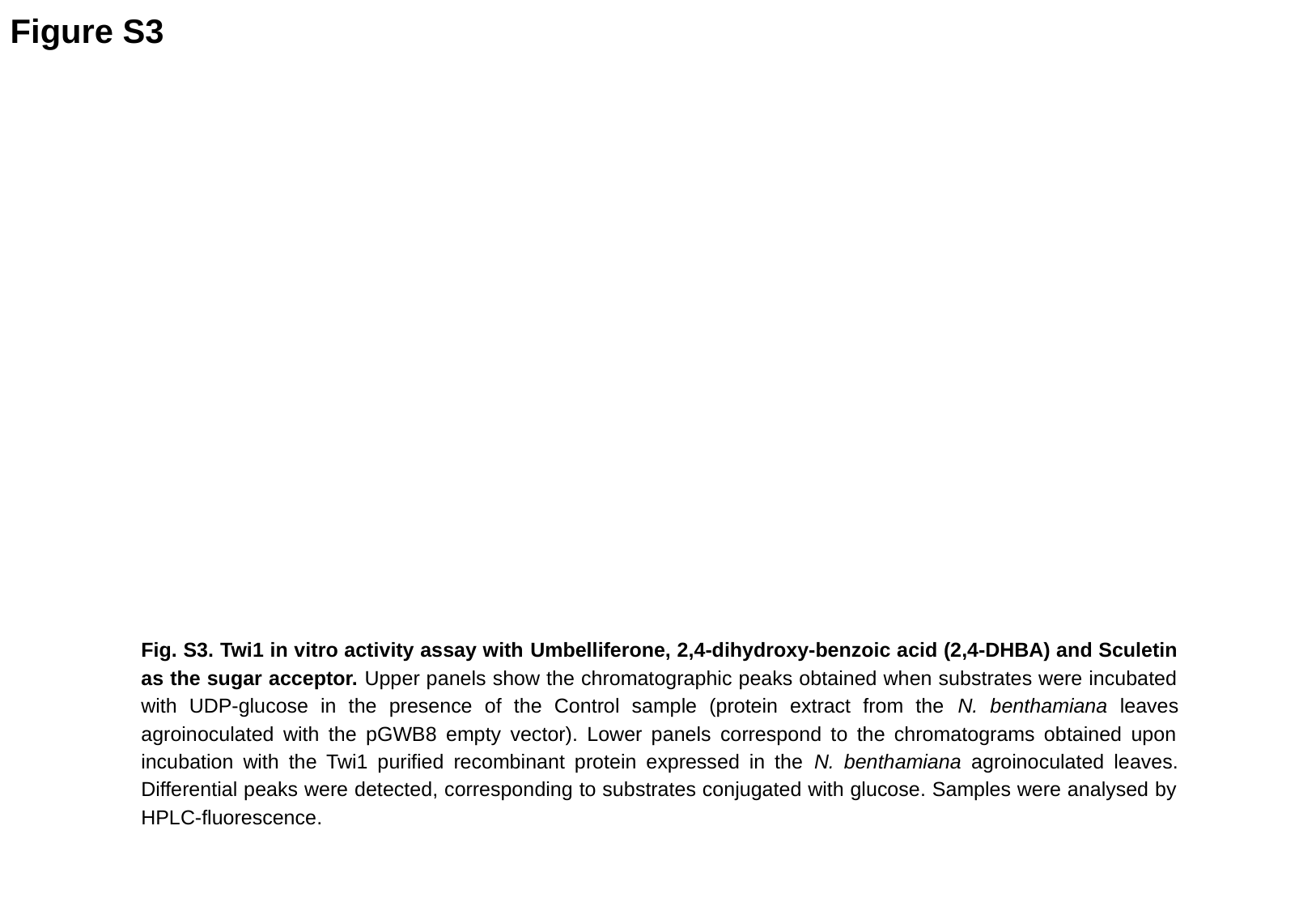

Figure S3
Fig. S3. Twi1 in vitro activity assay with Umbelliferone, 2,4-dihydroxy-benzoic acid (2,4-DHBA) and Sculetin as the sugar acceptor. Upper panels show the chromatographic peaks obtained when substrates were incubated with UDP-glucose in the presence of the Control sample (protein extract from the N. benthamiana leaves agroinoculated with the pGWB8 empty vector). Lower panels correspond to the chromatograms obtained upon incubation with the Twi1 purified recombinant protein expressed in the N. benthamiana agroinoculated leaves. Differential peaks were detected, corresponding to substrates conjugated with glucose. Samples were analysed by HPLC-fluorescence.
