# Supplementary material for: Tomato glycosyltransferase Twi1 plays a role in flavonoid glycosylation and defence against virus
Source: BMC Plant Biol. 2019 Oct 26;19:450. doi: 10.1186/s12870-019-2063-9 (PMC6815406; doi:10.1186/s12870-019-2063-9)
Supplement: Supplementary file 4 — Additional file 4: Figure S4. Twi1 enzyme activity towards 2,4,6-DHBA. [file 12870_2019_2063_MOESM4_ESM.pptx]

## Slide 1
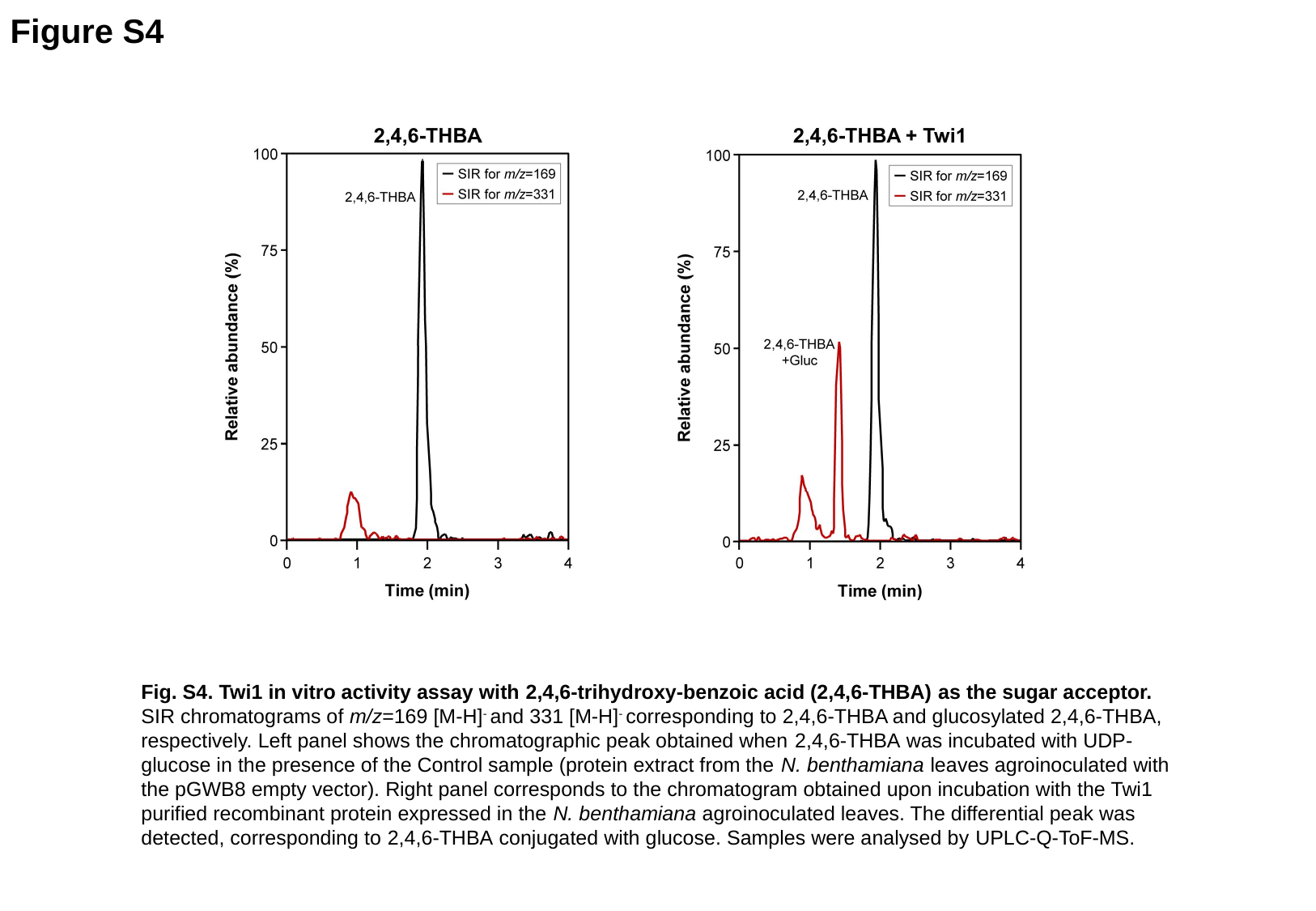

Figure S4
Fig. S4. Twi1 in vitro activity assay with 2,4,6-trihydroxy-benzoic acid (2,4,6-THBA) as the sugar acceptor. SIR chromatograms of m/z=169 [M-H]- and 331 [M-H]- corresponding to 2,4,6-THBA and glucosylated 2,4,6-THBA, respectively. Left panel shows the chromatographic peak obtained when 2,4,6-THBA was incubated with UDP-glucose in the presence of the Control sample (protein extract from the N. benthamiana leaves agroinoculated with the pGWB8 empty vector). Right panel corresponds to the chromatogram obtained upon incubation with the Twi1 purified recombinant protein expressed in the N. benthamiana agroinoculated leaves. The differential peak was detected, corresponding to 2,4,6-THBA conjugated with glucose. Samples were analysed by UPLC-Q-ToF-MS.
