# Supplementary material for: Tomato glycosyltransferase Twi1 plays a role in flavonoid glycosylation and defence against virus
Source: BMC Plant Biol. 2019 Oct 26;19:450. doi: 10.1186/s12870-019-2063-9 (PMC6815406; doi:10.1186/s12870-019-2063-9)
Supplement: Supplementary file 5 — Additional file 5: Figure S5. Disease severity of the transgenic and parental plants to TSWV virus. [file 12870_2019_2063_MOESM5_ESM.pptx]

## Slide 1
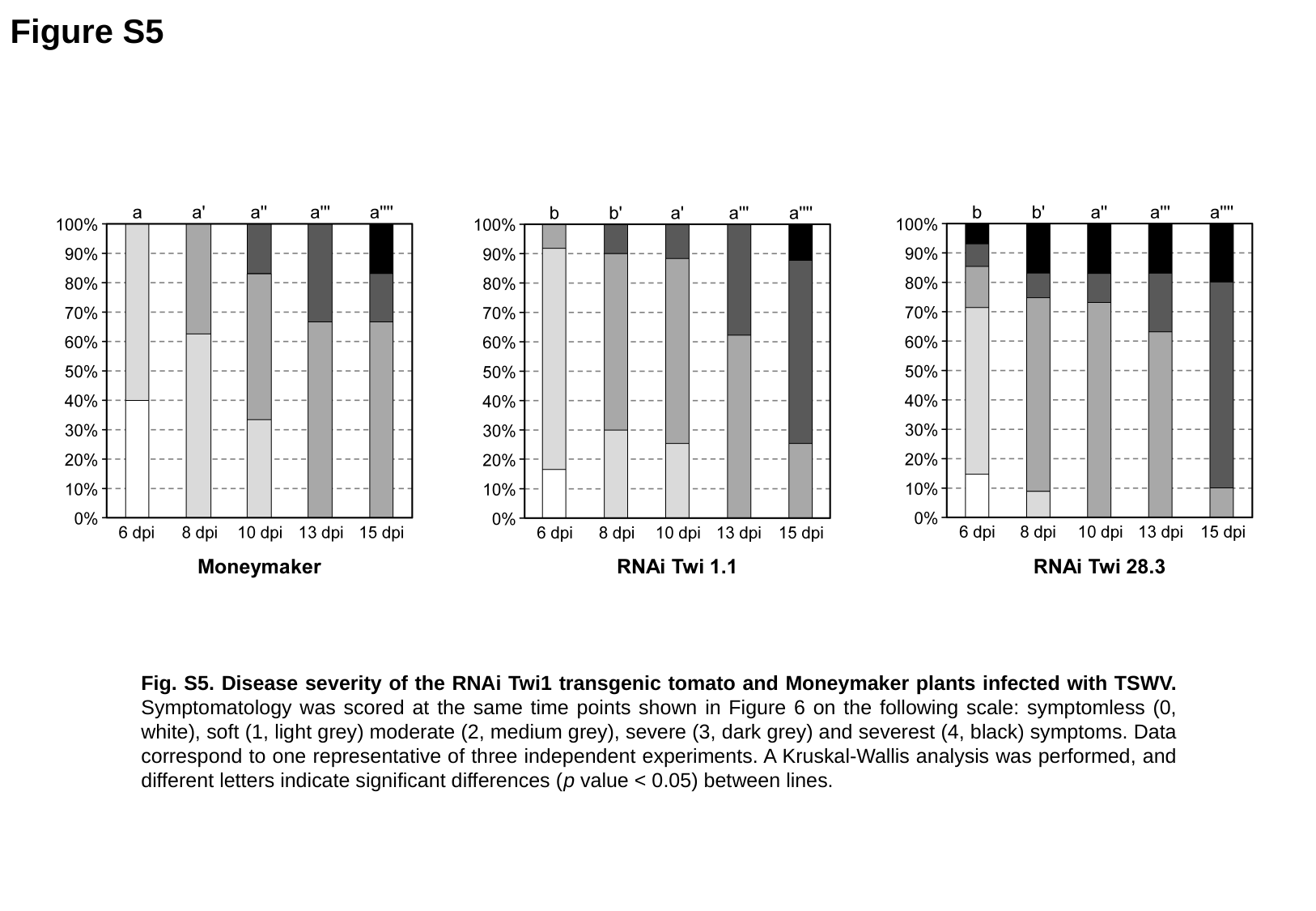

Figure S5
Fig. S5. Disease severity of the RNAi Twi1 transgenic tomato and Moneymaker plants infected with TSWV. Symptomatology was scored at the same time points shown in Figure 6 on the following scale: symptomless (0, white), soft (1, light grey) moderate (2, medium grey), severe (3, dark grey) and severest (4, black) symptoms. Data correspond to one representative of three independent experiments. A Kruskal-Wallis analysis was performed, and different letters indicate significant differences (p value < 0.05) between lines.
