# Supplementary material for: Tomato glycosyltransferase Twi1 plays a role in flavonoid glycosylation and defence against virus
Source: BMC Plant Biol. 2019 Oct 26;19:450. doi: 10.1186/s12870-019-2063-9 (PMC6815406; doi:10.1186/s12870-019-2063-9)
Supplement: Supplementary file 6 — Additional file 6: Figure S6. Twi1 gene expression transgenic tomato plants upon TSWV infection. [file 12870_2019_2063_MOESM6_ESM.pptx]

## Slide 1
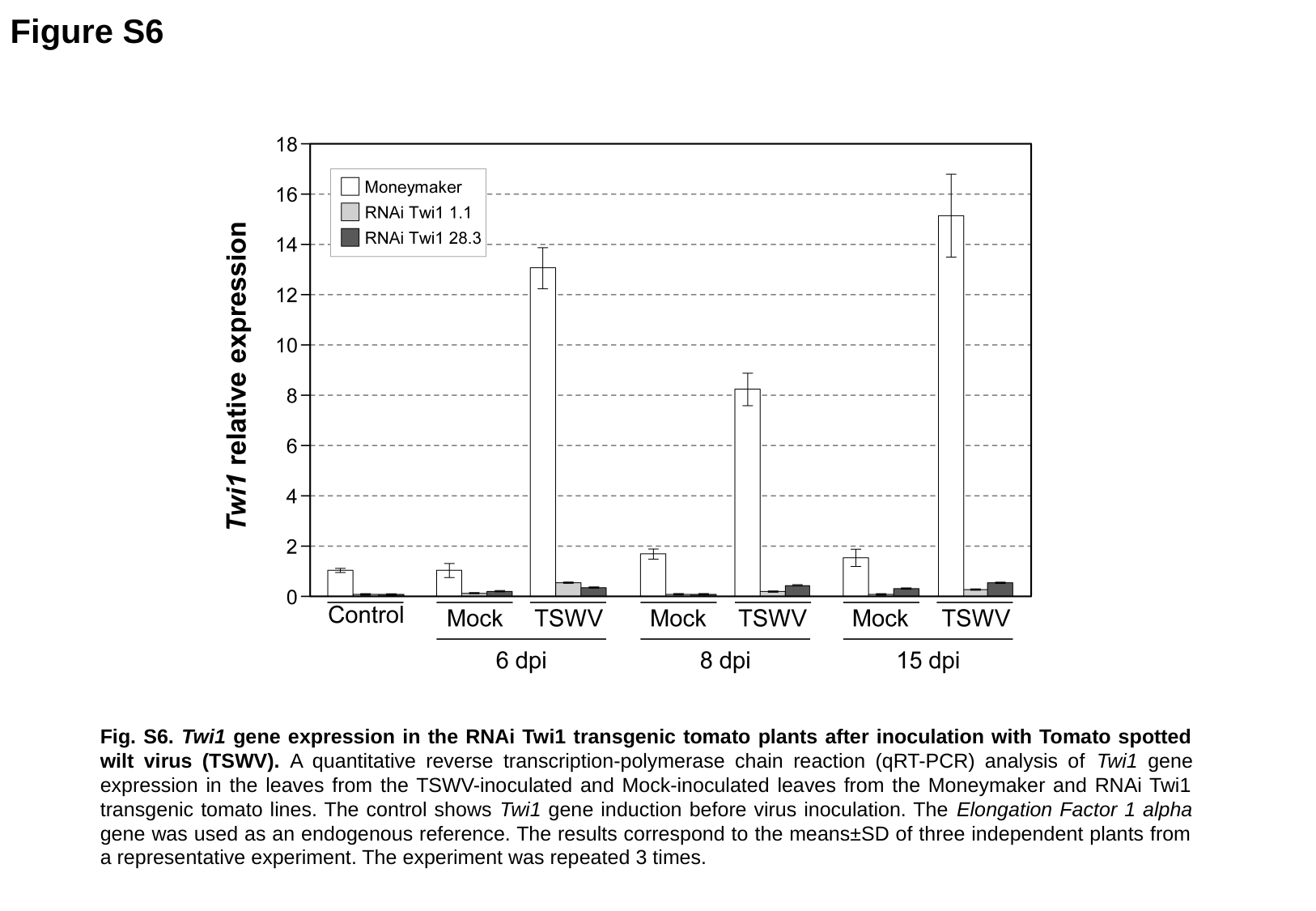

Figure S6
Fig. S6. Twi1 gene expression in the RNAi Twi1 transgenic tomato plants after inoculation with Tomato spotted wilt virus (TSWV). A quantitative reverse transcription-polymerase chain reaction (qRT-PCR) analysis of Twi1 gene expression in the leaves from the TSWV-inoculated and Mock-inoculated leaves from the Moneymaker and RNAi Twi1 transgenic tomato lines. The control shows Twi1 gene induction before virus inoculation. The Elongation Factor 1 alpha gene was used as an endogenous reference. The results correspond to the means±SD of three independent plants from a representative experiment. The experiment was repeated 3 times.
